# Understanding the interplay of compassion fatigue and moral resilience on moral distress in ICU nurses: a cross-sectional study

**DOI:** 10.3389/fpubh.2024.1402532

**Published:** 2024-10-28

**Authors:** Jin Yin, Lili Zhao, Na Zhang, Hui Xia

**Affiliations:** Department of Intensive Care Unit, The Affiliated Huaian First People's Hospital of Nanjing Medical University, Huai’an, China

**Keywords:** compassion fatigue, moral distress, moral resilience, ICU nurses, latent profiles, moderation effect, cross-sectional study

## Abstract

**Background:**

Intensive Care Unit (ICU) nurses frequently confront significant psychological challenges, including compassion fatigue, moral distress, and diminished moral resilience. These issues not only affect their well-being but also impact the quality of care provided to patients. The interplay of these factors is complex and not fully understood, particularly how compassion fatigue influences the relationship between moral resilience and moral distress.

**Objectives:**

To explore the complex interplay between compassion fatigue and moral distress among ICU nurses, and to elucidate how compassion fatigue influences the protective role of moral resilience against moral distress.

**Research design:**

A cross-sectional study was conducted using a nationwide random sample of ICU nurses in China. Latent profile analysis identified subgroups based on levels of compassion fatigue. Moderation analysis examined whether compassion fatigue moderated the association between moral resilience and moral distress.

**Results:**

Among 612 ICU nurses, latent profile analysis revealed three distinct groups with high, moderate, and low levels of compassion fatigue. Being female was protective against high compassion fatigue, while ages 30–49 yrs., lack of bachelor’s degree, and dissatisfaction with salary increased compassion fatigue risk. Moderation analysis showed compassion fatigue significantly moderated the relationship between moral resilience and moral distress. Nurses with higher compassion fatigue exhibited a stronger association between low moral resilience and high moral distress.

**Conclusion:**

Compassion fatigue and moral distress are interconnected phenomena among ICU nurses. Demographic factors like gender, age, education, and income satisfaction impact compassion fatigue risk. High compassion fatigue impairs moral resilience, exacerbating moral distress. Comprehensive interventions targeting both compassion fatigue and moral resilience, tailored to nurses’ demographic profiles, are needed to support this workforce.

## 1 Introduction

The existing literature on compassion fatigue, moral distress, and moral resilience provides a crucial foundation for understanding their impact among ICU nurses. Compassion fatigue stems from the intense emotional weariness healthcare workers experience due to prolonged exposure to stressful situations, a common occurrence in ICU settings (1, 2). Moral distress arises when nurses face ethical dilemmas, unable to act according to their professional values, a situation exacerbated in the complex and high-stakes environment of ICUs. Moral resilience refers to the capacity to maintain one’s integrity in the face of moral adversity, mitigating the effects of both compassion fatigue and moral distress (3, 4). Research suggests a dynamic interplay among these variables, where increased compassion fatigue can escalate moral distress by diminishing nurses’ moral resilience, highlighting the need for strategies that bolster resilience to improve nurse well-being and patient care outcomes. This enriched understanding sets the stage for our study, exploring how these factors interact and influence one another in the demanding ICU environment.

ICU nurses frequently confront substantial psychological hurdles stemming from moral distress, a condition that arises when they identify the ethically correct action but find themselves unable to act upon it, culminating in feelings of unease or psychological discomfort (3, 5). This form of distress is mainly instigated by ethical quandaries and decision-making conflicts encountered in clinical settings, eliciting adverse emotional reactions such as guilt, frustration, and an inclination to exit the nursing field (3, 4). Such situations often force nurses to administer care that contradicts their professional ethics or personal convictions, resulting in moral distress. This distress is associated with professional burnout and can negatively affect their standing in the profession and the caliber of care they provide (4). The consequences of moral distress in nursing can span various detrimental outcomes, impacting their mental health, professional behavior, and the overall efficiency of the healthcare system. Consequently, this distress can lead to a decrease in motivation and commitment to their role. This can adversely affect the quality of patient care (6). Nurses experiencing persistent moral distress might engage in withdrawal behaviors, distancing themselves from patients and peers alike. Such withdrawal can hinder the teamwork and communication vital for effective nursing care (7). One crucial organizational impact of moral distress is an increase in turnover rates, as nurses struggling to align their ethical convictions with their professional responsibilities might choose to resign, leading to staffing deficits and elevated costs associated with hiring and training new personnel (8). Indirectly, the effects of moral distress on nurses can also influence patient care. Nurses undergoing moral distress may show reduced engagement, potentially resulting in lower patient satisfaction and compromised care quality (9). Although existing literature extensively explores these phenomena individually, there is a lesser focus on how they interact dynamically within nursing environments. Our study seeks to bridge this gap by examining how compassion fatigue not only correlates with moral distress but also plays a moderating role in the interaction between moral resilience and moral distress. Moral resilience, defined as the ability to maintain one’s ethical integrity in the face of ethical challenges, can potentially mitigate the effects of moral distress. However, our study proposes that compassion fatigue can weaken this protective effect of moral resilience, thereby exacerbating the experience of moral distress. By explicitly addressing the moderating effect of compassion fatigue, deeper insights could be gained into the factors that influence moral distress and to identify potential levers for intervention to support ICU nurses more effectively.

Compassion fatigue experienced by Intensive Care Unit nurses encompasses the profound emotional and physical depletion arising from their work in highly stressful environments, such as ICUs, where they routinely encounter traumatic situations and witness intense patient suffering (2). This condition is a result of the cumulative psychological toll of providing care, as nurses deeply empathize with patients’ distress, leading to their own secondary traumatic stress (10–12).

Compassion fatigue among Intensive Care Unit nurses is characterized by significant emotional and physical exhaustion that stems from their work in high-stress conditions, where they frequently face traumatic events and observe severe patient distress (2). This syndrome develops from the accumulated emotional impact of caregiving, as nurses profoundly connect with the suffering of their patients, leading to secondary traumatic stress in themselves. However, existing studies have shown that some interventions can effectively address and mitigate compassion fatigue. These interventions can range from educational programs designed to help manage emotional stress, improvements in communication skills, the adoption of alternative scheduling for intensive care staff, to the practice of relaxation methods (13).

The experience of compassion fatigue and moral distress significantly impacts ICU nurses, adding their likelihood of leaving their jobs. These elements are interconnected in a complex and nuanced way, potentially exacerbating one another. Investigations have uncovered meaningful associations among secondary traumatic stress, burnout, and moral distress. Elevated instances of compassion fatigue in nurses correlate with higher moral distress levels, which subsequently heighten their inclination to resign (14). Furthermore, research has identified a positive correlation between moral distress and compassion fatigue among nursing staff, implying that the causes of moral distress might also lead to compassion fatigue. This suggests that approaches aimed at mitigating one issue could also address the other (15). The intensive care unit’s demanding environment, where nurses frequently face traumatic scenarios, can lead to both moral distress and compassion fatigue. These issues might intensify one another, as the emotional toll of compassion fatigue could hinder dealing with the ethical challenges that cause moral distress, and the reverse is also true. This reciprocal relationship highlights the critical need for interventions designed to tackle both problems simultaneously, aiming to improve ICU nurses’ welfare and retention (2).

Our study investigates the influence of demographic factors—specifically age, gender, education level, and salary satisfaction—on compassion fatigue among ICU nurses. Research indicates that younger and female nurses are often more susceptible to compassion fatigue due to less experience and greater emotional engagement in caregiving roles (16). Additionally, higher educational attainment is associated with reduced compassion fatigue, as more educated nurses possess better coping mechanisms and stress management strategies (17, 18). Moreover, dissatisfaction with salary exacerbates compassion fatigue, as financial stress can compound the emotional exhaustion of nursing duties (19, 20). Thus, our research hypothesizes that these demographic variables significantly impact the levels of compassion fatigue experienced by ICU nurses, guiding targeted interventions to mitigate its effects. This concise narrative establishes the rationale for our focus and sets the stage for exploring these relationships in our analysis.

Given the strong link between moral distress and compassion fatigue, it is evident that strategies for prevention and mitigation are essential. Such strategies could include training on identifying and managing both conditions, psychological support, fostering a supportive work culture, and establishing policies that encourage nurses to express their ethical concerns without fear of backlash. Considering that the compassion fatigue is preventable and intervenable, developing interventions to reduce compassion fatigue and simultaneously lower the moral distress is significant. The dynamic between compassion fatigue and moral distress among ICU nurses calls for an all-encompassing intervention strategy that focuses on both personal coping strategies and organizational support. By addressing these interrelated challenges, healthcare institutions can improve the work conditions for nurses, leading to better patient care and decreased turnover among nursing staff.

Latent profile analysis (LPA) is a sophisticated statistical technique used to identify subgroups within a population based on observed variables. In the context of our study on ICU nurses, LPA was chosen because it allows for the identification of distinct patterns or profiles of compassion fatigue, moral distress, and moral resilience that might not be apparent through other analytical methods. Theoretical support for the use of LPA in our study stems from the recognition that ICU nurses do not experience these phenomena uniformly; instead, they exhibit variations that likely form distinct profiles based on their professional experiences, coping mechanisms, and work environment characteristics.

From a theoretical perspective, the person-centered approach of LPA aligns with the nursing and psychological literature that suggests variability in how caregivers experience and respond to occupational stressors (21). For example, studies have shown that factors like years of experience, work environment, educational background, and personal resilience influence how nurses manage and react to compassion fatigue (16, 20, 22–24). By applying LPA, we aim to capture these nuances and provide a detailed examination of how different subgroups of nurses might benefit from tailored interventions. Moreover, the use of LPA supports the development of more targeted and effective strategies by identifying characteristic patterns among nurses, which can inform both preventative measures and interventions to enhance moral resilience and reduce the negative impacts of compassion fatigue and moral distress.

This study aims to fill this gap by:

Investigating the interplay between compassion fatigue and moral distress among ICU nurses.
Examining how compassion fatigue moderates the relationship between moral resilience (the capacity to sustain ethical integrity under stress) and moral distress.

To rigorously address these aims, we propose the following hypotheses:

*H1:* Compassion fatigue will moderate the relationship between moral resilience and moral distress among ICU nurses, such that higher levels of compassion fatigue will weaken the protective effect of moral resilience against moral distress.

*H2:* Reductions in compassion fatigue will strengthen the capacity of moral resilience to mitigate moral distress.

These hypotheses are formulated on the premise that compassion fatigue can alter the efficacy with which moral resilience counteracts the adverse effects of moral distress. Specifically, we anticipate that as compassion fatigue increases, the buffering role of moral resilience diminishes, leading to heightened moral distress. Conversely, lowering compassion fatigue is expected to enhance the effectiveness of moral resilience, thereby reducing the incidence and severity of moral distress experienced by nurses.

This investigation is crucial as it provides a theoretical foundation for developing interventions aimed at reducing moral distress through the management of compassion fatigue and enhancement of moral resilience. By exploring these dynamics, this research contributes significantly to the existing literature by delineating mechanisms that could inform more targeted and effective strategies to support ICU nurses in their high-stress roles.

## 2 Methods

### 2.1 Design

This was a cross-sectional study. We followed the guidelines recommended in the Strengthening the Reporting of Observational Studies in Epidemiology (STROBE) statement (25) when presenting our findings.

### 2.2 Study setting and sampling

The study recruited ICU nurses from 17 provinces nationwide across various regions to ensure a diverse and representative sample. Recruitment was facilitated through hospital administration offices, which assisted in identifying eligible participants based on the inclusion criteria: registered ICU nurses currently practicing and willing to participate in the study. Data collection took place from June to October 2023. The primary consideration for sample selection was the accessibility of the research subjects. We supplemented the sample using a snowball sampling method. Data were collected using a structured questionnaire tailored for this study, which was administered electronically via Wenjuanxing platform, a commonly used questionnaire collection website in China. Participants received a link to the questionnaire through their professional email addresses, provided by the hospital administrators. The questionnaire was designed to be completed within a median time of approximately 20 min. Before participating, all respondents were informed about the purpose of the study, the confidentiality of their responses, and their right to withdraw at any time without penalty. Informed consent was obtained electronically; participants were required to tick a box indicating their voluntary agreement to participate before they could proceed to the questionnaire. This consent process was reviewed and approved by the institutional review board of The Affiliated Huai’an first People’s Hospital of Nanjing Medical University, Huai’an, China.

### 2.3 Inclusion and exclusion criteria

The inclusion criteria for the study subjects comprised: (1) working in a general or specialized ICU setting; (2) engaging in clinical duties; (3) providing informed consent. The exclusion criteria for the research subjects included: (1) nursing interns; (2) nurses borrowed from other departments to work in the ICU; (3) with excessively short completion times. In our study, we excluded questionnaires with excessively short completion times to ensure the quality and reliability of the data collected. Specifically, completion times that were less than one-third of the median completion time were considered excessively short, based on recommendations from past literature that suggest such responses are likely to be unreliable due to insufficient attention or engagement (26, 27). In our study, the median completion time was 20 min, responses completed in less than approximately 7 min were excluded. This approach is consistent with similar methodologies employed in healthcare research to enhance data validity.

### 2.4 Instrument with validity and reliability

#### 2.4.1 Demographic characteristics

Demographic characteristics such as gender, age, marital status, ICU working time, educational level, professional title, Income (per month/CNY), satisfaction with income.

#### 2.4.2 Professional quality of life scale, ProQOL

Compassion satisfaction is measured alongside compassion fatigue and burnout using the Professional Quality of Life Scale (ProQOL). This scale is integral to our study as it assesses both the positive and negative aspects of caregiving. Compassion satisfaction refers to the positive feelings derived from competent caregiving, and it is essential to include it to provide a holistic view of the ICU nurses’ experiences. The Professional Quality of Life Scale (ProQOL) was developed by Dr. Beth Hudnall Stamm. It is designed to measure the levels of compassion satisfaction and compassion fatigue (which includes both burnout and secondary traumatic stress) experienced by professionals working in caring and helping roles. The construct validity of the ProQOL has been confirmed through several studies using confirmatory factor analysis and other statistical methods. These studies often highlight the scale’s ability to accurately measure the constructs of compassion satisfaction, burnout, and secondary traumatic stress as distinct yet related aspects of professional quality of life. The scale has also been adapted and validated in different cultural contexts, ensuring its applicability across diverse populations (28). The content construct of the scale is 0.82. The ProQOL demonstrates high internal consistency and reliability across its subscales. Studies have reported Cronbach’s alpha values that indicate good to excellent reliability for the compassion satisfaction, burnout, and secondary traumatic stress components. The scale’s Cronbach’s *α* is 0.91 (28). Cronbach’s α of the three sub-scales of ProQoL are 0.82, 0.73, and 0.76 (29). The Professional Quality of Life Scale (ProQOL) is widely utilized to assess the negative and positive effects of helping others who experience suffering and trauma. The scale comprises three subscales: Compassion Satisfaction, Burnout, and Secondary Traumatic Stress, each with 10 items. Compassion Satisfaction measures the pleasure derived from being able to do one’s work effectively. Burnout and Secondary Traumatic Stress assess feelings of hopelessness and difficulties in dealing with work, and the work-related, secondary exposure to extremely stressful events, respectively. Respondents rate each item on a 5-point Likert scale from 1 (never) to 5 (very often), reflecting how frequently they experience the associated feelings.

#### 2.4.3 Moral distress scale-revised, MDS-R

The Moral Distress Scale-Revised (MDS-R) is an instrument designed to measure the degree of moral distress experienced by healthcare professionals in various clinical settings. The MDS-R is a revised version of the original Moral Distress Scale, which was developed by Corley et al. (30) in the early 2000s. The revision was undertaken to improve the scale’s reliability and validity and to make it more applicable across different healthcare disciplines and settings. The revised scale includes scenarios that are common sources of moral distress for healthcare professionals, developed by Hamric (31), such as feeling pressured to provide care that is not in the best interest of the patient, witnessing inadequate patient care due to a lack of resources, and working with colleagues who are not competent. Respondents rate each item based on both the frequency with which they experience the described situation and the intensity of the distress it causes them. This dual-focus approach helps to capture the multifaceted nature of moral distress, providing insights into not only how often healthcare professionals encounter ethically challenging situations but also the severity of the distress these situations cause. The CVI of Chinese edition is 0.909. The scale’s Cronbach’s *α* is 0.879 (30). The reliability and validity of the Chinese version of MDS-R were confirmed to be satisfactory, exhibiting a Cronbach’s alpha of 0.879 and a test–retest reliability of 0.802 (32). The Moral Distress Scale-Revised (MDS-R) is designed to measure the intensity and frequency of moral distress experienced by healthcare professionals. This scale consists of 21 items that describe situations known to evoke moral distress such as following orders that go against what one believes is right. Each item is scored based on both the frequency (from 0 ‘never’ to 4 ‘very frequently’) and intensity (from 0 ‘none’ to 4 ‘great’) of the distress experienced, allowing for a nuanced assessment of moral distress. The MDS-R helps identify specific areas where ethical conflicts are most pronounced, guiding interventions to reduce distress.

#### 2.4.4 Rushton moral resilience scale, RMRS

The Rushton Moral Resilience Scale (RMRS), developed by Heinze et al. (33) is a validated instrument developed to measure moral resilience among healthcare professionals. The scale quantifies the ability of individuals to maintain or restore their moral integrity in the face of moral adversity. The RMRS includes 16 items that are rated on a 5-point Likert scale from 0 (never) to 4 (always). The scale measures several dimensions of moral resilience, such as the capacity for positive moral emotions, the ability to maintain moral agency, and the persistence of moral efficacy in challenging situations. The RMRS is a validated tool, demonstrating strong psychometric properties in various healthcare settings, and provides insights into how individuals navigate complex ethical environments and sustain their commitment to ethical practice. The RMRS was developed through a comprehensive process that included item development, expert review, focus groups with healthcare professionals, and extensive psychometric testing. The RMRS demonstrated acceptable validity and reliability, with a final structure that includes dimensions of responses to moral adversity, personal integrity, moral efficacy, and relational integrity. The CVI of each item ranged from 0.820–1.000. The RMRS exhibits strong overall reliability (*α* = 0.84) and has shown convergent validity with the Connor Davidson Resilience Scale-10 as well as criterion validity with the Maslach Burnout Inventory Human Services Survey (34).

### 2.5 Data analysis

To begin with, descriptive statistics were performed using IBM SPSS 26. To ensure the appropriateness of the statistical tests used, we first assessed whether the data was normally distributed. The Shapiro–Wilk test was utilized to determine normality. For variables where the Shapiro–Wilk test returned a *p*-value greater than 0.05, indicating normal distribution, parametric tests were employed. These included independent *t*-tests for comparing two groups and ANOVA (Analysis of Variance) for multiple group comparisons. Variables that did not exhibit normal distribution, as indicated by Shapiro–Wilk test *p*-values less than 0.05, were analyzed using non-parametric tests. The Mann–Whitney *U* test was used for two-group comparisons, and the Kruskal-Wallis test was applied when comparing more than two groups. Accordingly, parametric or non-parametric tests were employed based on these results. The representation of continuous variables depends on their adherence to a normal distribution. If the data is normally distributed, the mean ± standard deviation is employed. Alternatively, if the data deviates from a normal distribution, the median and interquartile range are utilized for representation. In the case of categorical variables, representation is achieved through frequency and percentages.

To address potential common method bias, which can occur due to the use of self-report measures collected at a single point in time, we conducted a principal component analysis (PCA) on all the study variables using scales to measure. This methodological step is crucial for identifying any single underlying structure that might artificially inflate relationships among variables. PCA was chosen due to its effectiveness in revealing the dimensionality of the data and its ability to highlight the variance explained by a potential common method factor.

We conducted Latent Profile Analysis (LPA) on our dataset using Mplus 8.4, starting with a one-class model and incrementally exploring multiple models (35–37). Model fit was evaluated using Akaike Information Criterion (AIC), Bayesian Information Criterion (BIC), Adjusted BIC, entropy, bootstrap likelihood ratio test, and Lo–Mendell–Rubin likelihood ratio test, focusing on interpretability and practical significance. Lower AIC, BIC, and aBIC values indicated better model fit, while higher entropy values suggested greater classification accuracy. Significance in the bootstrap and Lo–Mendell–Rubin tests supported the model with optimal latent classes.

Next, we analyzed the formation of latent profiles for nurses’ compassion fatigue. Using these profiles, we conducted univariate analysis of demographic information. Non-parametric tests were employed due to non-normal distribution in some groups. Two logistic regressions were performed to investigate the effects of demographic variables on compassion fatigue levels, confirming significant predictors with *p* < 0.05.

Lastly, we examined the moderating role of compassion fatigue on the relationship between moral resilience and moral distress using hierarchical regression. This analysis grouped the low and moderate compassion fatigue profiles together to assess their collective moderation effect on the continuous variable of moral resilience. Changes in R-squared values and the Durbin-Watson statistic helped validate the absence of autocorrelation and the significance of moderation effects. This phase utilized Python 3.12.2 to generate moderation effect plots, providing practical insights into how different levels of compassion fatigue affect the resilience-distress relationship among ICU nurses.

## 3 Results

### 3.1 Descriptive statistics

We surveyed a total of 887 ICU nurses for this study, of which 645 completed the questionnaire. This results in a response rate of approximately 72.7%. After excluding some invalid ones (such as those with excessively short completion times or where the same options were consistently selected across multiple scales), the final number of valid questionnaires was 612. The basic information of the participants is presented in Table 1. The majority of participants were female (89.4%), aged 18–29 years (45.9%) or 30–39 years (46.2%), married (61.4%), had ≤5 years of work experience (50.7%), held a bachelor’s degree (76.3%), possessed a primary nurse title (64.5%), had a monthly income of 4,001–8,000 RMB (52.3%), and expressed dissatisfaction with their salary (55.1%).

**Table 1:** Characteristics of participants.

Variables	*N*	Percentage (%)
Gender		
Male	65	10.6%
Female	547	89.4%
Age		
18–29	281	45.9%
30–39	283	46.2%
≥40	48	7.9%
Marital status		
Married	376	61.4%
Single	236	38.5%
ICU working time		
≤5	310	50.7%
6–10	171	27.9%
11–15	98	16.0%
≥16	33	5.4%
Education level		
≤College	127	20.8%
Undergraduate	467	76.3%
≥Postgraduate	18	2.9%
Professional title		
Primary title	395	64.5%
Middle title	197	32.2%
Advanced title	20	3.3%
Income (per month/ CNY)		
≤4,000	85	13.9%
4,001–8,000	320	52.3%
8,001–12,000	176	28.8%
>12,000	31	5.1%
Satisfaction with income		
Yes	275	44.9%
No	337	55.1%

The principal component analysis extracted one factor that accounted for 28.777% of the total variance among the variables, which is below the often-cited threshold of 50% used to indicate a significant common method bias (38). This result suggests that while some common method variance is present, it does not dominate the responses, thereby not excessively influencing the relationships among the study variables. Table 2 provided the Means, Standard Deviations, and Correlations of the core variables of this study.

**Table 2:** Means, standard deviations, and correlations.

Variable	M	SD	1	2	3
1. Compassion fatigue	92.59	16.25	1	0.168^**^	−0.293^**^
2. Moral distress	141.39	99.42	0.168^**^	1	−0.098^*^
3. Moral resilience	9.42	1.01	−0.293^**^	−0.098^*^	1

^*^P<0.05; ^**^P<0.01.

### 3.2 Latent profile analysis

**Figure 1:**
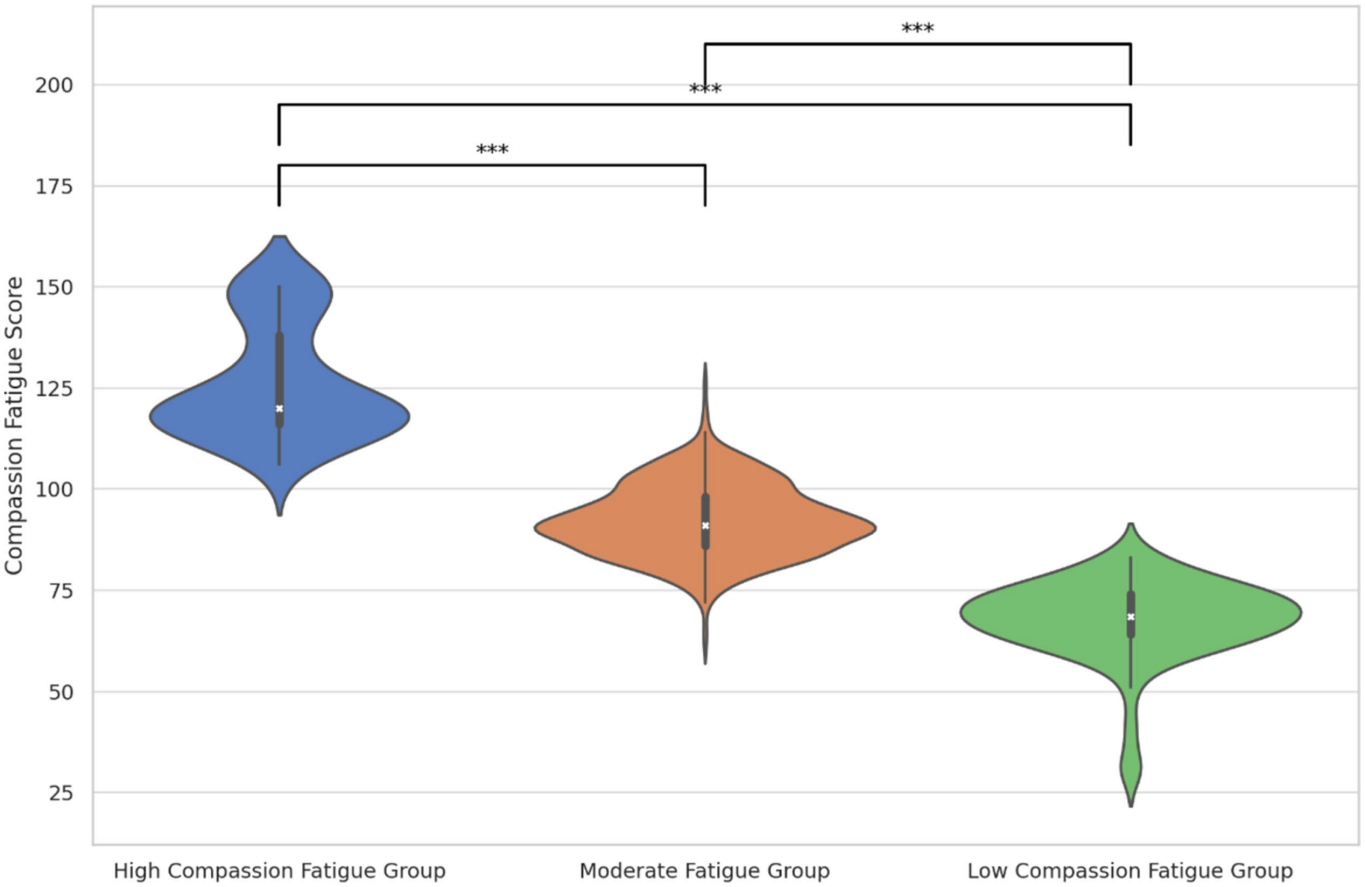
The violin plot of three latent profiles of compassion fatigue.

**Figure 2:**
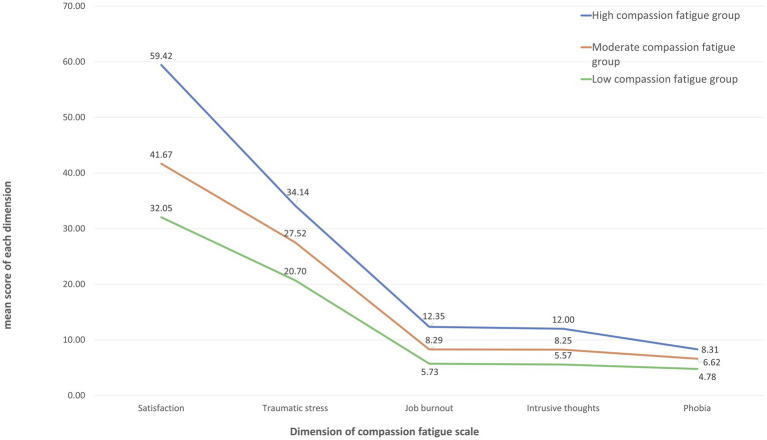
Mean score in 5 dimensions of compassion fatigue questionnaire in 3 latent profiles of ICU nurses.

Table 3 details the model fit metrics (AIC, BIC, aBIC) which decreased as profiles increased, highlighting improved model fit. Significant results from the bootstrap likelihood ratio test and Lo–Mendell–Rubin test supported both the 3-profile and 4-profile models, with the 3-profile model showing optimal entropy, indicating its superior fit. Thus, we adopted the 3-profile model to represent the diversity in ICU nurses’ compassion fatigue. Figures 1, 2 illustrate the distribution and mean scores across the profiles, labeled as High, Moderate, and Low Compassion Fatigue Groups, following a pattern from high to low across five dimensions. Notably, the mean scores in each dimension of ICU nurses’ compassion fatigue follow the pattern: high > moderate > low. Based on practical implications in clinical settings, we have designated the groups as the High Compassion Fatigue Group, Moderate Compassion Fatigue Group, and Low Compassion Fatigue Group, respectively.

**Table 3:** Model fit results.

Model	AIC	BIC	aBIC	Entropy	LMR	BLRT	Latent class proportions
1	15848.345	15892.512	15860.764				1
2	15225.782	15296.450	15245.653	0.945	0.000	0.000	0.89/0.11
3	14874.826	14971.995	14902.149	0.908	0.0000	0.0000	0.79/0.12/0.09
4	14697.920	14821.589	14732.694	0.901	0.0001	0.0001	0.75/0.10/0.13/0.02
5	14613.328	14763.497	14655.554	0.913	0.1618	0.1689	0.11/0.71/0.13/0.01/0.02

### 3.3 Univariate analysis and logistic regression

Table 4 presents the results of the chi-square tests, indicating that significant differences were observed among different groups of ICU nurses with compassion fatigue only in terms of satisfaction with their salary. No differences were detected in the remaining variables. Table 5 presents the comparisons of latent profiles of continuous variables of ICU nurses. We established two logistic regression models to examine the impact of demographic characteristics on the grouping of compassion fatigue. The results can be found in Table 6. In the comparison between the High vs. Moderate and Low groups, being female was identified as a protective factor for compassion fatigue. In the comparison between the High and Moderate vs. Low groups, being in the age group of 30–49 years was recognized as a risk factor for compassion fatigue relative to the 18–29 age group, while holding a bachelor’s degree was identified as a protective factor against compassion fatigue, and dissatisfaction with salary was highlighted as a risk factor for compassion fatigue.

**Table 4:** Comparison of different latent profiles of general demographic data of nurses.

Variable	Latent profile			Chi-square value	*P*
	High	Moderate	Low		
Variables					
Gender					
Male	11 (6.1)	48 (51.7)	6 (7.2)	5.053	0.080
female	46 (50.9)	439 (435.3)	62 (60.8)		
Age					
18–29	22 (26.2)	230 (223.6)	29 (31.2)	5.584†	0.223
30–39	31 (26.4)	215 (225.2)	37 (31.4)		
≥40	4 (4.1)	42 (38.2)	2 (5.3)		
Marital status					
Married	35 (35.0)	303 (299.2)	38 (41.8)	1.011	0.602
Single	22 (22.0)	184 (187.8)	30 (26.2)		
ICU working time					
≤5	25 (28.9)	249 (246.7)	36 (34.4)	3.159^†^	0.795
6–10	16 (15.9)	135 (136.1)	20 (19.0)		
11–15	11 (0.1)	77 (78.0)	10 (10.9)		
≥16	5 (3.1)	26 (26.3)	2 (3.7)		
Education level					
≤College	8 (11.8)	97 (101.1)	22 (14.1)	7.105^†^	0.107
Undergraduate	48 (43.5)	374 (371.6)	45 (51.9)		
≥Postgraduate	1 (1.7)	16 (14.3)	1 (2.0)		
Professional title					
Primary title	36 (36.8)	307 (314.3)	52 (43.9)	8.379^†^	0.079
Middle title	17 (18.3)	164 (156.8)	16 (21.9)		
Advanced title	4 (1.9)	16 (15.9)	0 (2.2)		
Income (per month/ CNY)					
≤4,000	6 (7.9)	64 (67.6)	15 (9.4)	6.746^†^	0.338
4,001–8,000	30 (29.8)	257 (254.6)	33 (35.6)		
8,001–12,000	16 (6.4)	144 (140.1)	16 (19.6)		
>12,000	5 (2.9)	22 (24.7)	4 (3.4)		
Satisfaction with income					
Yes	28 (25.6)	228 (218.8)	19 (30.6)	9.038	0.011
No	29 (31.4)	259 (268.2)	49 (37.4)		

^†Calculate by Fisher’s exact test.^

**Table 5:** Comparisons of latent profiles of continuous variables of ICU nurses.

Variables	*T* value	*Post hoc* comparison	*P*
1. Compassion fatigue	292.135	1–2-3^†^	<0.05
2. Moral distress	38.225	1–2-3	<0.05
3. Moral resilience	36.191	1–2-3	<0.05

^†^“1–2-3” indicates that there are statistically significant differences (in *P*<0.05 level) in pairwise comparisons among latent profile 1, 2, and 3.

**Table 6:** Results of logistic regression.

Variable	Odds ratio with 95% CI	
	High VS. moderate and low	High and moderate VS. low
Gender (ref: male)		
Female	0.421 (0.197, 0.899)	0.747 (0.299, 1.865)
Age (ref: 18–20)		
30–39	0.584 (0.234, 1.456)	3.363 (1.487, 7.609)
≥40	5.586 (0.518, 60.189)	0.873 (0.087, 8.782)
Marital status (ref: married)		
Single	0.742 (0.355, 1.548)	1.505 (0.767, 2.956)
ICU working time (ref: ≤5)		
6–10	0.918 (0.408, 2.064)	0.862 (0.421, 1.766)
11–15	0.616 (0.225, 1.692)	0.924 (0.358, 2.383)
≥16	0.144 (0.020, 1.016)	2.863 (0.302, 27.139)
Education level (ref: ≤College)		
Undergraduate	0.626 (0.276, 1.421)	0.510 (0.276, 0.944)
≥Postgraduate	2.337 (0.218, 25.037)	0.302 (0.032, 2.866)
Professional title (ref: Primary title)		
Middle title	1.520 (0.673, 3.436)	0.401 (0.186, 0.862)
Advanced title	0.411 (0.070, 2.423)	0.000 (0.000, 0.000)
Income (per month/ CNY) (ref:≤4000)		
4,001–8,000	0.878 (0.331, 2.329)	0.610 (0.292, 1.274)
8,001–12,000	1.144 (0.373, 3.505)	0.743 (0.299, 1.847)
>12,000	0.469 (0.092, 2.403)	3.277 (0.739, 14.533)
Satisfaction with income (ref: Yes)		
No	1.198 (0.660, 2.173)	2.577 (1.395, 4.761)

### 3.4 The moderating effect of with different latent profiles

**Figure 3:**
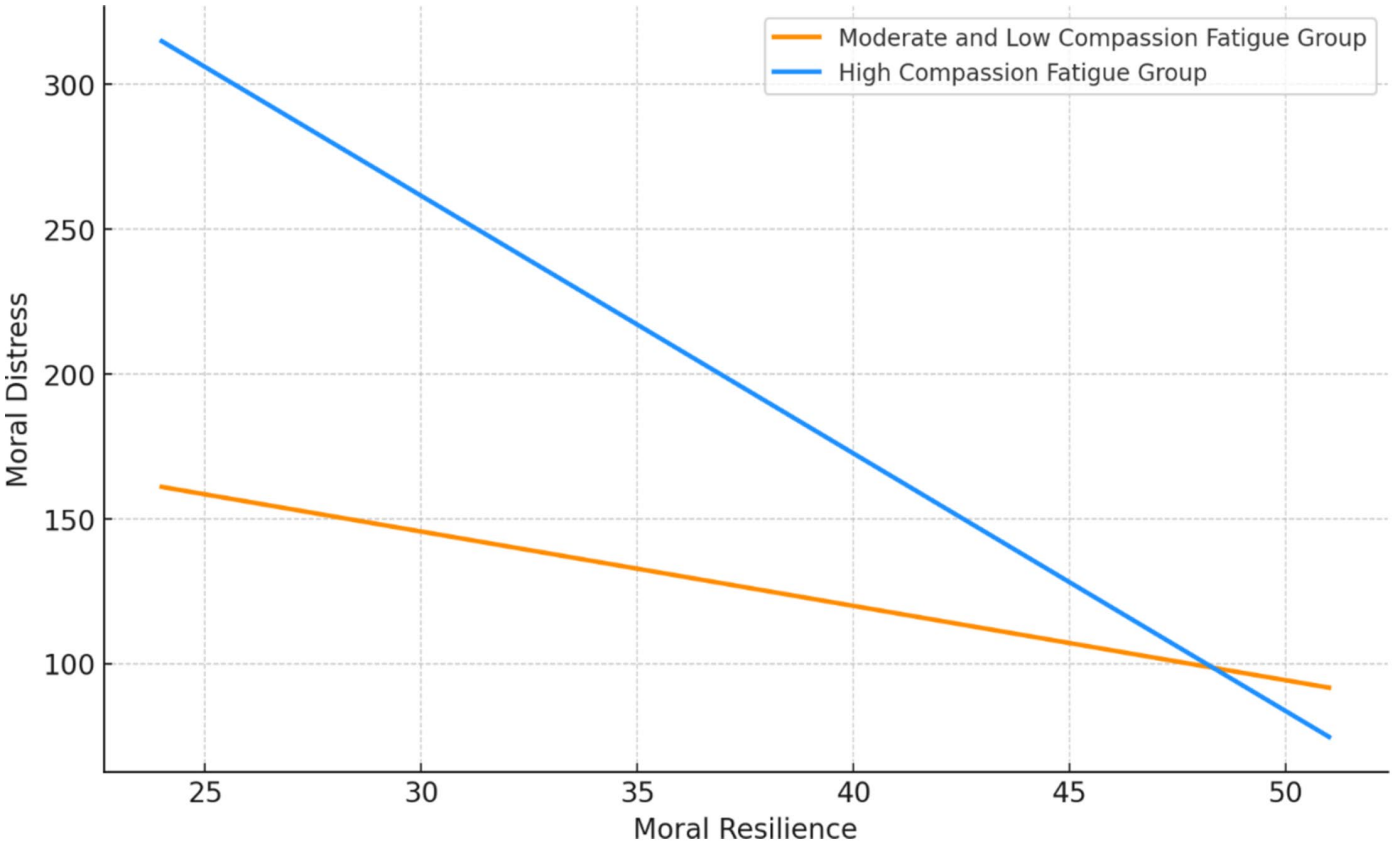
The different effect from moral resilience to moral distress in different latent profile groups.

The hierarchical regression analysis yielded noteworthy findings. Upon the inclusion of the interaction term between the independent variables and the moderating variable, the adjusted R-squared value notably increased from the original 0.074 to 0.488. This substantial increase in adjusted R-squared is statistically significant (*F* = 493.724, *p* < 0.001), thus indicating that the relationship between moral resilience and moral distress is influenced by the presence of compassion fatigue. Moreover, the Durbin-Watson statistic of 2.041, which is proximate to the ideal value of 2, suggests the absence of significant collinearity between the independent variables and the moderating variable. Consequently, it can be inferred that nurses experiencing higher levels of compassion fatigue are more likely to exhibit elevated levels of moral distress, as indicated in Figure 3.

## 4 Discussion

The primary objective of this study was to explore the interplay between compassion fatigue, moral distress, and moral resilience among ICU nurses and to examine how compassion fatigue moderates the relationship between moral resilience and moral distress. By conducting this research, we aimed to provide insights into how these psychological dynamics affect ICU nurses’ professional lives and identify potential interventions to mitigate these effects. This study sought to fill gaps in the current understanding of how the cumulative burden of compassion fatigue could influence nurses’ capacity to manage moral distress through resilience. The results highlight several key findings that contribute to our understanding of these complex relationships, providing a foundation for the development of targeted strategies to support nursing professionals in high-stress environments.

The findings of this study provide valuable insights into the interplay between compassion fatigue and moral distress among ICU nurses. The latent profile analysis revealed three distinct groups of nurses experiencing varying levels of compassion fatigue: high, moderate, and low.

Notably, the univariate analysis and logistic regression results identified several demographic factors associated with increased compassion fatigue. Being female was recognized as a protective factor for high compassion fatigue when compared to the moderate and low groups. This finding is intriguing as it diverges from previous research that indicated women might be more vulnerable to compassion fatigue, likely due to societal expectations and gender roles influencing their emotional responses (39–41). Additionally, nurses aged 30–39 years exhibited a higher risk of compassion fatigue compared to their younger counterparts. This age group often faces significant personal and professional responsibilities, potentially contributing to increased stress and emotional strain. However, although we want to draw a conclusion between the increased age and high level of compassion fatigue, the statistical result was not significant in the ≥40 years old group. It is plausible that the insufficient sample size (*n* = 48) of this group may have hindered its ability to achieve statistical power. Comparing our results with prior research reveals inconsistencies about the impact of age on compassion fatigue, with earlier studies reporting higher levels among younger nurses than their more experienced counterparts (16, 42, 43).

Interestingly, holding a bachelor’s degree emerged as a protective factor against high compassion fatigue. This finding underscores the importance of higher education in equipping nurses with the necessary knowledge and skills to cope with the demands of their profession effectively. Similar with previous studies, the higher educational levels in nurses may correlate with lower levels of compassion fatigue (44–46). Nurses with higher level of education may have advanced coping strategies and mechanisms for dealing with stress and trauma encountered in their work (44–46). In other words, higher education comes better access to resources, including knowledge of where to find psychological support, understanding of mental health, and awareness of self-care strategies (47, 48).

Moreover, dissatisfaction with salary was identified as a contributing factor to higher compassion fatigue, suggesting the potential impact of financial stress and perceived inadequate compensation on nurses’ emotional well-being. Interestingly, the actual income levels did not show a significant correlation with compassion fatigue, highlighting that it is the perception of income adequacy, rather than the absolute income amount, that may influence emotional health (49–51). This distinction underscores the importance of exploring how satisfaction with wages affects compassion fatigue in future research, which could inform health policy and salary allocation in hospitals. These findings suggest that enhancing satisfaction with income, even without changes to wage distribution, could potentially reduce compassion fatigue among nurses, offering a valuable strategy for healthcare management to consider.

The moderation analysis revealed a significant interaction between moral resilience, moral distress, and compassion fatigue. Nurses experiencing higher levels of compassion fatigue exhibited a stronger association between moral resilience and moral distress (14, 15, 52). This finding suggests that compassion fatigue may potentially hinder nurses’ ability to maintain moral resilience in the face of ethical challenges, exacerbating the impact of moral distress. This finding can generate practical implications for nurse managers. The intervention focus on the moral distress is limited. Lowering the compassion fatigue can simultaneously reduce the moral distress level. This indicate that when developing intervention to reduce the level of moral distress, the component of the intervention such as peer support, professional counseling could be also considered (48).

These results highlight the complex interplay between compassion fatigue and moral distress, emphasizing the need for comprehensive interventions that address both phenomena simultaneously. Strategies aimed at reducing compassion fatigue, such as fostering self-care practices, promoting work-life balance, and providing emotional support resources, could potentially mitigate the negative effects of moral distress. Conversely, interventions focused on enhancing moral resilience, including ethical decision-making training, open communication channels, and a supportive organizational culture, may alleviate the emotional burden associated with compassion fatigue.

Furthermore, the identification of demographic risk factors for compassion fatigue underscores the importance of tailoring interventions to specific nursing populations. For instance, targeted support and resources could be provided to female nurses, those in the 30–49 age range, and individuals expressing dissatisfaction with their compensation.

This study extends the understanding of compassion fatigue among ICU nurses by elucidating the role of demographic factors in its prevalence and intensity. By applying latent profile analysis, our research not only confirms the heterogeneity in compassion fatigue experiences among nurses but also underscores the significant influence of age, gender, education, and salary satisfaction. Theoretically, this work enriches existing healthcare literature by integrating a nuanced perspective on how individual differences affect psychological outcomes in high-stress environments. Our findings suggest a need to consider these variables in the development of personalized interventions aimed at reducing compassion fatigue.

While our study provides important insights, it is not without limitations. The cross-sectional design limits the ability to infer causality between demographic factors and compassion fatigue. Additionally, the reliance on self-reported data may introduce response bias. Future research could benefit from a longitudinal design to track changes over time and employ mixed methods to corroborate self-reported data with observational or qualitative data. Further studies might also explore additional variables, such as organizational culture or specific work-related stressors, that could interact with demographic factors to influence compassion fatigue.

This study contributes to a deeper understanding of the interplay between compassion fatigue and moral distress in the ICU nursing context. The findings highlight the need for comprehensive interventions that address both phenomena while considering individual demographic factors. By implementing targeted strategies to alleviate compassion fatigue and enhance moral resilience, healthcare organizations can improve the well-being of their nursing staff, ultimately leading to better patient outcomes and a more sustainable healthcare workforce.

## 5 Theoretical and practical implications

### 5.1 Theoretical implications

Our findings contribute to the body of knowledge on psychological stress in nursing by delineating the complex relationships between compassion fatigue, moral distress, and moral resilience. This study underscores the theory that compassion fatigue can significantly undermine the protective factors of moral resilience, providing a nuanced understanding of how chronic exposure to stress affects healthcare professionals’ ethical decision-making and psychological well-being.

### 5.2 Practical implications

The results underscore the necessity for targeted interventions aimed at reducing compassion fatigue among ICU nurses. Practical applications of these findings could include the development of resilience training programs, regular psychological assessments, and the introduction of support structures that specifically address the sources of moral distress. Healthcare institutions could implement these strategies to foster a more supportive environment that actively mitigates the risks associated with compassion fatigue.

### 5.3 Moderating role of compassion fatigue

Further analysis reveals that compassion fatigue significantly moderates the relationship between moral resilience and moral distress. This moderation suggests that even robust moral resilience may falter under the weight of severe compassion fatigue. Our findings suggest that interventions aimed at reducing compassion fatigue may not only improve individual coping mechanisms but also enhance the effectiveness of resilience-building strategies already in place.

### 5.4 Limitations

This study’s primary limitation is its cross-sectional design, which restricts our ability to infer causation from the observed relationships. Additionally, the reliance on self-reported measures may introduce response biases, potentially skewing the reported levels of compassion fatigue, moral distress, and resilience. Future research should consider longitudinal approaches to validate these findings and explore the dynamics of these relationships over time.

### 5.5 Suggestions for future studies

To build on our findings, future research could explore the causal relationships through experimental or longitudinal studies. Expanding the scope to include diverse nursing populations and different healthcare settings would also enrich the understanding of how contextual factors influence compassion fatigue, moral resilience, and moral distress. Such studies could help to tailor interventions more precisely to the needs of specific nurse demographics or specialties.

## 6 Conclusion

This study highlights the complex relationship between compassion fatigue, moral distress, and moral resilience among ICU nurses, identifying three distinct groups with varying levels of compassion fatigue influenced by factors such as gender, age, education, and salary satisfaction. The analysis shows that higher compassion fatigue correlates with increased moral distress, suggesting that it diminishes nurses’ moral resilience against ethical challenges. Addressing both compassion fatigue and moral distress through targeted interventions, like fostering self-care, promoting work-life balance, and enhancing moral resilience with ethical training and supportive organizational culture, is essential. Such tailored strategies not only improve nurse well-being but also enhance patient care and contribute to a more sustainable healthcare system.

## Data Availability

The raw data supporting the conclusions of this article will be made available by the authors, without undue reservation.
